# Complications of pelvic and acetabular fractures in 1331 morbidly obese patients (BMI ≥ 40): a retrospective observational study from the National Trauma Data Bank

**DOI:** 10.1186/s13037-018-0172-2

**Published:** 2018-08-29

**Authors:** James T. Carson, Sabin G. Shah, Gezzer Ortega, Sorawut Thamyongkit, Erik A. Hasenboehler, Babar Shafiq

**Affiliations:** 1 Department of Orthopaedic Surgery, Howard University College of Medicine, Washington, DC, USA; 2 Department of Orthopaedic Surgery, University of California Irvine, Orange, CA USA; 3 Outcomes Research Center, Department of Surgery, Howard University College of Medicine, Washington, DC, USA; 4 Department of Orthopaedic Surgery, The Johns Hopkins University School of Medicine, 601 N Caroline St, Fl 5, Baltimore, MD 21205 USA

**Keywords:** Acetabular fracture, Complications, Morbid obesity, Pelvic fracture

## Abstract

**Background:**

There have been no large-scale epidemiological studies of outcomes and perioperative complications in morbidly obese trauma patients who have sustained closed pelvic ring or acetabular fractures. We examined this population and compared their rate of inpatient complications with that of control patients.

**Methods:**

We retrospectively reviewed the records of patients treated for closed pelvic ring or acetabular fracture, aged 16–85 years, with Injury Severity Scores ≤15 from the National Trauma Data Bank Research Dataset for the years 2007 through 2010. The primary outcome of interest was rate of in-hospital complications. Secondary outcomes were length of hospital stay and discharge disposition. Unadjusted differences in complication rates were evaluated using Student t tests and Chi-squared analyses. Multiple logistic and Poisson regression were used to analyze binary outcomes and length of hospital stay, respectively, adjusting for several variables. Statistical significance was defined as *p* < 0.05.

**Results:**

We included 46,450 patients in our study. Of these patients, 1331 (3%) were morbidly obese (body mass index ≥40) and 45,119 (97%) were used as controls. Morbidly obese patients had significantly higher odds of complication and longer hospital stay in all groups considered except those with pelvic fractures that were treated operatively. In all groups, morbidly obese patients were more likely to be discharged to a skilled nursing/rehabilitation facility compared with control patients.

**Conclusions:**

Morbidly obese patients had higher rates of complications and longer hospital stays and were more likely to be discharged to rehabilitation facilities compared with control patients after pelvic ring or acetabular fracture.

## Background

In the United States, obesity is a public health crisis, with its high prevalence remaining stable over the past decade [1, 2]. The Centers for Disease Control and Prevention reports that 34.9% of all U.S. adults (approximately 78 million) and 16.9% of all U.S. adolescents (approximately 12.5 million) are considered obese [2]. Obesity is associated with higher rates of surgical complications, including difficulty with anesthesia, postoperative infections, and thromboembolic disease [3–6].

Obese and morbidly obese patients (body mass index [BMI] ≥30 and BMI ≥40, respectively) [7, 8] with pelvic fractures have been shown to have longer operative times [9], greater estimated intraoperative blood loss [10], and higher rates of wound infection, wound dehiscence, loss of reduction, iatrogenic nerve injury, pneumonia, and decubitus ulcers [11–13]. There is an absence of large-scale epidemiological studies on outcomes and perioperative complications in morbidly obese trauma patients who have sustained closed pelvic ring or acetabular fractures. The steady rise of obesity, coupled with the magnitude of potential complications associated with these injuries, prompted us to further study this subset of morbidly obese orthopaedic trauma patients. The purpose of this study was to analyze the incidence of postoperative complications among morbidly obese trauma patients who sustained closed pelvic fractures and to compare it with non–morbidly obese patients who underwent comparable treatment. We hypothesize that there will be a higher incidence of in-hospital complications in morbidly obese patients with closed pelvic and acetabular fractures treated operatively or nonoperatively compared with non–morbidly obese patients.

## Methods

We conducted a retrospective analysis using the National Trauma Data Bank (NTDB) Research Dataset for 2007 through 2010. This study was based on data from a publicly available database and therefore was exempt from institutional review board approval. For this type of study, formal consent is not required. The NTDB contains data from more than 1.9 million trauma admissions at more than 900 U.S. trauma centers throughout the country and is maintained by the American College of Surgeons. Detailed information about NTDB data collection procedures is available on the NTDB website [14]. It contains information pertaining to inpatients admitted through emergency departments and links data by unique, nonidentifying incident keys.

**Fig. 1:**
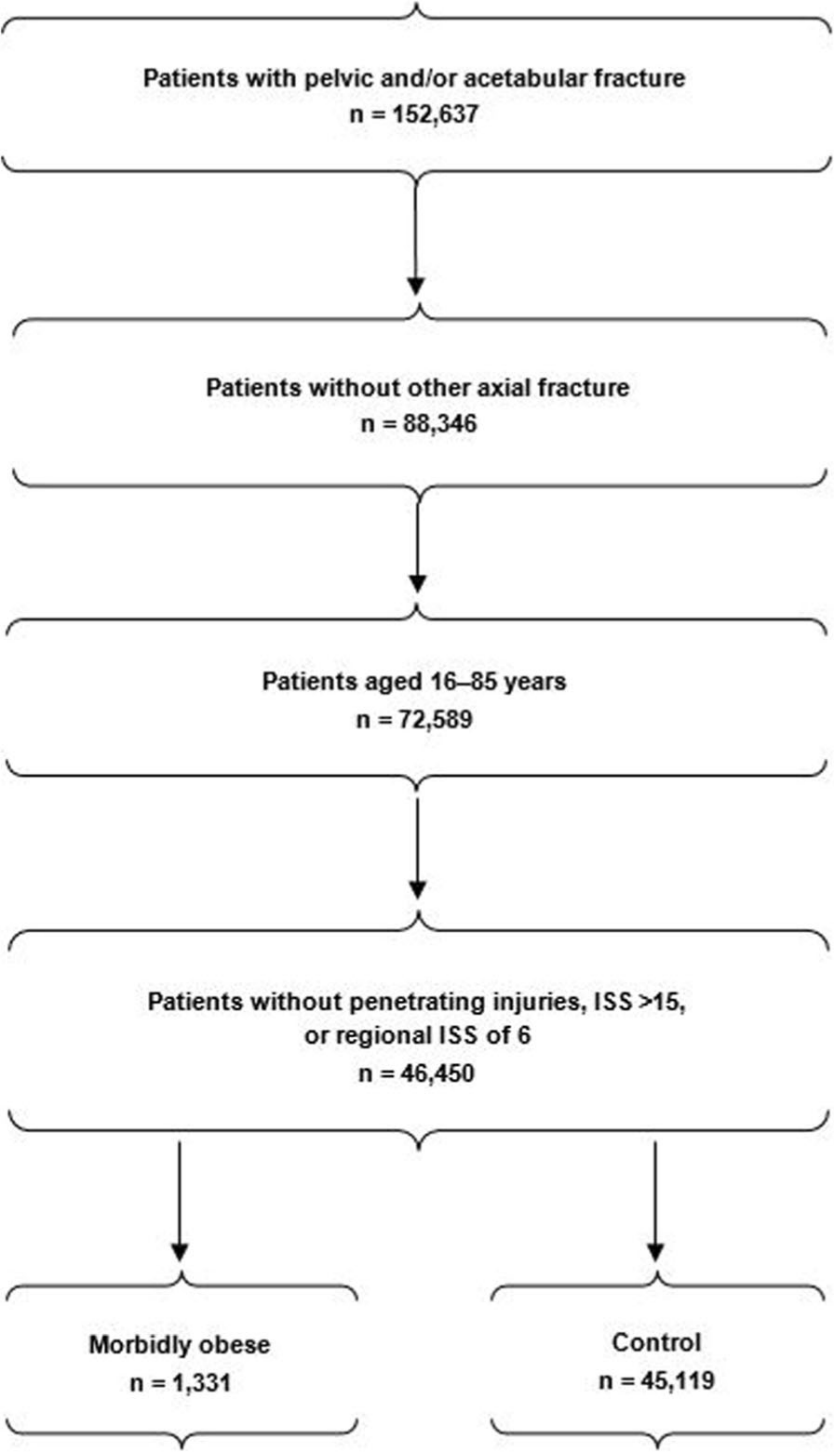
Criteria for patient selection into the study

We used the following inclusion criteria to select patients: (1) closed pelvic and/or acetabular fracture (*International Classification of Diseases, Ninth Revision* [15] codes 808.0, 808.2, 808.4, 808.41, 808.42, 808.43, 808.49, 808.8); and (2) age 16 years through 85 years. Exclusion criteria were (1) age younger than 16 years or older than 85 years; (2) penetrating trauma; (3) insufficient data for analysis; (4) Injury Severity Score (ISS) > 15 (5) regional ISS of 6; and (6) phalanx or spine fractures. Patients with phalanx and spine fractures were eliminated because their procedure codes overlap with those of pelvic and acetabular fractures. Patients with ISS > 15 were excluded because they fall into the major (or polytrauma) trauma category, confounding the effect of pelvic and acetabular fractures alone [16] Patients with a regional ISS score of 6 were also excluded because they are deemed to have untreatable (fatal) injuries [16, 17]. All patients who met the inclusion criteria were placed in 1 of 2 groups: (1) morbidly obese and (2) not morbidly obese (“control”). Morbid obesity was determined by ICD-9 code (278.00 or 278.01) or database comorbidity listing for obesity (the NTDB distinguishes patients with BMI ≥40 as morbidly obese [18]). Details of the selection process are provided in Fig. 1. We identified 152,637 patients with closed pelvic and/or acetabular fractures. After applying our exclusion criteria, 46,450 (64%) patients remained. Of these, 1331 (2.87%) were morbidly obese.

We evaluated the following parameters: diagnosis, fracture management, patient characteristics (age, sex, health insurance type, most common mechanism of trauma and comorbidities), fracture management (operative vs. nonoperative), ISS, complications, presence of hypotension on admission, hospital teaching status, hospital trauma level, length of stay (LOS), and discharge disposition. We evaluated the following complications: acute renal failure, acute respiratory distress syndrome, bleeding, cardiac arrest, coagulopathy, decubitus ulcer, deep venous thrombosis (DVT), myocardial infarction, surgical site infection (SSI) (superficial or deep), organ or deep space infection, pulmonary embolus, stroke, sepsis, and wound disruption.

For data analysis, the groups were stratified by injury type as follows: pelvic fracture (with no acetabular fracture), acetabular fracture (with no pelvic fracture), and both pelvic and acetabular fractures. These groups were further stratified by whether they received operative or nonoperative treatment.

### Statistical analysis

To determine differences in groups for continuous variables, we used 2-tailed t tests. Categorical variables were examined using Chi-squared tests. Fisher exact test was used for categorical variables when the numbers were too small to allow use of the Chi-squared test. Bivariate analysis was used to show raw percentages of outcomes. Multiple logistic and Poisson regression were used to analyze binary outcomes and LOS, respectively, adjusting for age, sex, race/ethnicity, insurance status, injury characteristics (ISS, Abbreviated Injury Score, mechanism), presence/absence of hypotension on admission, hospital factors (trauma level, teaching status), and treatment type (open reduction and internal fixation, internal fixation, external fixation). All analyses were performed with Stata, version 11.0 (StataCorp, College Station, Texas). Statistical significance was defined as *p* < 0.05.

## Results

A total of 46,450 patients met our inclusion criteria. Demographic characteristics of control and morbidly obese patients are shown in Table 1. Morbidly obese patients were significantly younger (47 vs. 52 years, *p* < 0.05) and more likely to be female (51% vs. 43%) compared with controls. They were more likely to have a high-energy mechanism of injury such as motor vehicle collision, and they required operative treatment more frequently than the control patients. Morbidly obese patients had a higher overall complication rate when treated operatively or nonoperatively compared with the control group. This difference was significant (*p* < 0.05) in all but the operative pelvic fracture group. Moreover, morbid obesity was associated with approximately twice the odds of having a complication when operative or nonoperative treatment occurred. These odds were significant in all but the operative pelvic fracture group (Table 2).

**Table 1:** Characteristics of 46,450 patients treated for pelvic and/or acetabular fracture, National trauma data bank research dataset, 2007–2010

Characteristic	Control^a^ (*N* = 45,119)		Morbidly Obese (*N* = 1331)		*p* Value
	*N*	%	*N*	%	
Fracture type					
Pelvic only	21,238	47.1	324	24.3	< 0.001
Acetabular only	15,904	35.2	761	57.2	
Pelvic and acetabular	7977	17.7	246	18.5	
Male sex	25,496	56.5	654	49.1	< 0.001
Race					
White	33,654	75	959	72	< 0.001
Black	4898	11	206	15	
Hispanic	706	1.6	10	0.75	
Other	3858	8.5	84	6.3	
Unknown	2003	4.4	72	5.4	
Mechanism of trauma					
Motor vehicle collision	14,559	32	776	58	< 0.001
Fall	18,766	42	336	25	
Struck pedestrian	2168	5.4	33	2.7	
Injury Severity Score					
0–8	21,454	47.5	536	40.3	< 0.001
9–15	23,665	52.4	795	59.7	
Age group, years					
16–24	6789	15.0	167	12.5	< 0.001
25–44	11,000	24.4	458	34.4	
45–64	11,973	26.5	429	32.2	
65–85	15,357	34.0	277	20.8	
Procedure type					
External fixation	490	4.56	28	4.82	0.771
Internal fixation	299	2.78	18	3.10	0.653
ORIF	10,283	95.7	561	96.6	0.319
Needing operative fixation					
All	10,745	23.8	581	43.6	< 0.001
Pelvic fracture	1401	6.60	44	13.6	< 0.001
Acetabular fracture	6605	41.5	415	54.5	< 0.001
Both fractures	2739	34.3	122	49.6	< 0.001

ORIF, open reduction and internal fixation

^a^Patients with body mass index values < 40

**Table 2:** Analysis of inpatient complications by Fracture type in the nonoperative and operative groups by Obesity Status

Complication by Fracture Type	Nonoperative Group (*N* = 35,124)					Operative Group (*N* = 11,326)				
	Control^a^, % (*N*)	Morbidly Obese, % (*N*)	*p* Value	OR^b,c^ (95% CI)	*p* Value	Control^a^, % (*N*)	Morbidly Obese, % (*N*)	*p* Value	OR^b,c^ (95% CI)	*p* Value
Overall complications										
Pelvic only	3.83 (759)	11.8 (33)	< 0.001	2.98 (2.03–4.38)	< 0.001	8.42 (118)	20.5 (9)	0.006	2.20 (0.93–5.17)	0.071
Acetabular only	4.06 (378)	8.38 (29)	< 0.001	2.12 (1.41–3.20)	< 0.001	6.00 (396)	11.3 (47)	< 0.001	1.99 (1.43–2.78)	< 0.001
Pelvic and acetabular	5.35 (280)	11.3 (14)	0.004	2.07 (1.14–3.77)	0.017	10.9 (299)	23.8 (29)	< 0.001	2.41 (1.52–3.84)	< 0.001
Acute renal failure										
Pelvic only	0.80 (159)	3.57 (10)	< 0.001	4.20 (2.16–8.15)	< 0.001	1.14 (16)	2.27 (1)	0.493	1.12 (0.12–10.9)	0.922
Acetabular only	0.73 (68)	1.45 (5)	0.133	1.99 (0.77–5.14)	0.156	0.58 (38)	1.69 (7)	0.006	4.13 (1.73–9.86)	0.001
Pelvic and acetabular	0.94 (49)	3.23 (4)	0.011	3.17 (1.07–9.38)	0.037	1.13 (31)	5.74 (7)	< 0.001	8.43 (3.11–22.8)	< 0.001
Acute respiratory distress syndrome										
Pelvic only	0.43 (85)	1.79 (5)	0.001	3.03 (1.17–7.85)	0.023	1.00 (14)	2.27 (1)	0.412	0.99 (0.06–15.7)	0.995
Acetabular only	0.56 (52)	1.73 (6)	0.006	2.94 (1.21–7.14)	0.017	0.71 (47)	1.45 (6)	0.094	2.04 (0.85–4.89)	0.112
Pelvic and acetabular	0.36 (19)	1.61 (2)	0.028	4.48 (0.97–20.7)	0.055	1.13 (31)	3.29 (4)	0.035	2.56 (0.82–8.03)	0.107
Bleeding										
Pelvic only	0.19 (37)	1.43 (4)	< 0.001	7.15 (2.44–21.0)	< 0.001	0.79 (11)	0 (0)	0.555	NA	
Acetabular only	0.25 (23)	0 (0)	0.354	NA		0.30 (20)	0.48 (2)	0.527	1.73 (0.38–7.84)	0.478
Pelvic and acetabular	0.38 (20)	0 (0)	0.491	NA		0.69 (19)	0.82 (1)	0.87	1.06 (0.12–9.27)	0.956
Cardiac arrest with CPR										
Pelvic only	0.21 (42)	0 (0)	0.441	NA		0.29 (4)	2.27 (1)	0.027	6.10 (0.14–252)	0.341
Acetabular only	0.26 (24)	0.58 (2)	0.26	3.11 (0.69–14.0)	0.14	0.08 (5)	0.48 (2)	0.011	9.89 (1.61–60.7)	0.013
Pelvic and acetabular	0.46 (24)	0 (0)	0.45	NA		0.18 (5)	0.82 (1)	0.132	2.43 (0.24–24.6)	0.451
Coagulopathy										
Pelvic only	0.26 (51)	0.36 (1)	0.743	1.30 (0.17–9.72)	0.8	0.43 (6)	0 (0)	0.664	NA	
Acetabular only	0.26 (24)	0.58 (2)	0.26	1.72 (0.39–7.64)	0.477	0.20 (13)	0 (0)	0.366	NA	
Pelvic and acetabular	0.40 (21)	0 (0)	0.48	NA		0.26 (7)	0 (0)	0.576	NA	
Decubitus ulcer										
Pelvic only	0.34 (68)	2.86 (8)	< 0.001	7.08 (3.30–15.2)	< 0.001	0.93 (13)	2.27 (1)	0.37	1.86 (0.17–20.4)	0.613
Acetabular only	0.43 (40)	0.58 (2)	0.682	1.44 (0.35–3.72)	0.882	0.59 (39)	1.69 (7)	0.007	2.12 (1.17–3.83)	0.013
Pelvic and acetabular	0.73 (38)	2.42 (3)	0.032	2.68 (0.75–9.56)	0.129	1.10 (30)	4.92 (6)	< 0.001	4.61 (1.72–12.4)	0.002
Deep surgical site infection										
Pelvic only	NA	NA	NA	2.40 (0.73–7.85)	0.147	0.14 (2)	0 (0)	0.802	1.63 (0.32–8.28)	0.557
Acetabular only	NA	NA	NA	1.28 (0.30–5.50)	0.742	0.05 (3)	0.24 (1)	0.105	2.89 (1.24–6.72)	0.014
Pelvic and acetabular	NA	NA	NA	2.18 (0.65–7.39)	0.209	0.11 (3)	0 (0)	0.715	2.97 (1.49–5.95)	0.002
DVT/thrombophlebitis										
Pelvic only	0.36 (72)	1.07 (3)	0.053			2.21 (31)	4.55 (2)	0.308	NA	
Acetabular only	0.66 (61)	0.87 (3)	0.635			1.54 (102)	3.37 (14)	0.005	NA	
Pelvic and acetabular	0.86 (45)	2.42 (3)	0.068			3.10 (85)	9.84 (12)	< 0.001	NA	
Myocardial infarction										
Pelvic only	0.29 (58)	0.36 (1)	0.842	1.22 (0.17–8.93)	0.845	0 (0)	2.27 (1)	< 0.001	NA	
Acetabular only	0.18 (17)	0.58 (2)	0.104	4.71 (0.99–22.4)	0.052	0.17 (11)	0.48 (2)	0.147	4.96 (0.97–25.3)	0.054
Pelvic and acetabular	0.25 (13)	0 (0)	0.579	NA		0.40 (11)	0.82 (1)	0.484	0.99 (0.07–14.7)	0.996
Organ/deep space infection										
Pelvic only	1.26 (250)	3.57 (10)	0.001	2.36 (1.22–4.57)	0.011	3.28 (46)	11.36 (5)	0.004	2.84 (0.93–8.66)	0.067
Acetabular only	1.26 (117)	2.02 (7)	0.215	1.72 (0.78–3.83)	0.179	1.39 (92)	1.93 (8)	0.372	1.35 (0.64–2.85)	0.427
Pelvic and acetabular	1.68 (88)	4.84 (6)	0.008	2.89 (1.19–6.97)	0.018	3.10 (85)	6.56 (8)	0.035	1.85 (0.85–4.05)	0.127
Pulmonary embolism										
Pelvic only	0.27 (53)	0.36 (1)	0.773	1.32 (0.18–9.65)	0.785	1.28 (18)	0 (0)	0.449	NA	
Acetabular only	0.44 (41)	1.45 (5)	0.008	3.11 (1.19–8.17)	0.21	1.17 (77)	2.17 (9)	0.072	1.95 (0.05–4.01)	0.069
Pelvic and acetabular	0.42 (22)	0.81 (1)	0.515	1.41 (0.18–11.2)	0.747	1.53 (42)	4.10 (5)	0.029	2.34 (0.87–6.33)	0.092
Stroke/CVA										
Pelvic only	0.07 (14)	0 (0)	0.657	NA		0.14 (2)	0 (0)	0.802	NA	
Acetabular only	0.06 (6)	0.29 (1)	0.128	4.38 (0.44–43.5)	0.207	0.05 (3)	0 (0)	0.664	NA	
Pelvic and acetabular	0.08 (4)	0 (0)	0.758	NA		0.37 (10)	0.82 (1)	0.427	1.92 (0.22–16.9)	0.555
Superficial SSI										
Pelvic only	NA	NA	NA			0.29 (4)	2.27 (1)	0.143	8.12 (0.89–74.2)	0.064
Acetabular only	NA	NA	NA			0.26 (17)	0.48 (2)	0.311	1.88 (0.43–8.15)	0.401
Pelvic and acetabular	NA	NA	NA			0.33 (9)	2.46 (3)	0.013	7.65 (2.04–28.6)	0.003
Systemic sepsis										
Pelvic only	0.29 (57)	1.07 (3)	0.017	3.62 (1.10–11.9)	0.034	1.07 (15)	0 (0)	0.49	NA	
Acetabular only	0.45 (42)	1.16 (4)	0.062	2.45 (0.83–6.27)	0.106	0.42 (28)	1.20 (5)	0.024	3.18 (1.17–8.66)	0.023
Pelvic and acetabular	0.38 (20)	1.61 (2)	0.034	3.01 (0.65–14.1)	0.161	0.84 (23)	4.10 (5)	< 0.001	4.40 (1.52–12.73)	0.006
Wound disruption										
Pelvic only	NA	NA	NA			0.07 (1)	0 (0)	0.859	NA	
Acetabular only	NA	NA	NA			0. 06 (4)	0.24 (1)	0.181	6.10 (0.51–73.69)	0.155
Pelvic and acetabular	NA	NA	NA			0.29 (8)	0 (0)	0.55	NA	

*CI* confidence interval, *CPR* cardiopulmonary resuscitation, *CVA* cerebrovascular accident, *DVT* deep venous thrombosis, *NA* not applicable, *OR* odds ratio, *SSI* surgical site infection

^a^Patients with body mass index values < 40

^b^NA signifies that there was no significant difference between the groups, and too much colinearity existed to calculate an actual OR

^c^Adjusted for age, sex, race/ethnicity, insurance status, injury characteristics (Injury Severity Score, Abbreviated Injury Score, mechanism), presence/absence of hypotension on admission, hospital factors (trauma level, teaching status), and treatment type (open reduction with internal fixation, internal fixation, or external fixation)

In addition, morbidly obese patients had longer hospital stays than control patients for all injury and treatment types. This difference was significant for all categories except for operative treatment of pelvic fractures. Finally, morbidly obese patients were more likely to be sent to a skilled nursing or rehabilitation facility on discharge (*p* < 0.001) (Table 3).

**Table 3:** Secondary outcome measures of 46,450 patients treated for pelvic and/or acetabular fractures

Outcome Measure	Nonoperative Group					Operative Group				
	Control^a^ (*N* = 34,374)		Morbidly Obese (*N* = 750)		*p* Value	Control (*N* = 10,745)		Morbidly Obese (*N* = 581)		*p* Value
	%	Mean (CI)	%	Mean (CI)		%	Mean (CI)	%	Mean (CI)	
Length of hospital stay, days										
Pelvic only		5.3 (5.2–5.4)		7.5 (6.5–8.6)	< 0.001		11 (10–11)		13 (10–16)	0.055
Acetabular only		5.8 (5.7–6.0)		8.0 (7.2–8.9)			9.3 (9.1–9.4)		12 (11–13)	< 0.001
Pelvic and acetabular		6.4 (6.2–6.6)		10 (8.3–12)			11 (11–12)		15 (13–17)	
Discharge disposition					< 0.001					< 0.001
Home										
Pelvic only	47		36			62		40		
Acetabular only	59		50			68		45		
Pelvic and acetabular	46		27			54		36		
Skilled nursing/rehabilitation										
Pelvic only	46		61			36		58		
Acetabular only	30		44			30		53		
Pelvic and acetabular	46		70			45		64		
Other/unknown										
Pelvic only	6.9		3.6			1.7		2.3		
Acetabular only	11		6.8			1.5		1.7		
Pelvic and acetabular	7.9		2.5			1.6		0		

*CI* confidence interval, *OR* odds ratio

^a^Patients with body mass index values < 40

Poisson regression analysis was used to determine the incidence rate ratios (IRRs) comparing LOS for morbidly obese and control patients in each of the injury and treatment types. This regression model controlled for multiple factors and represents how LOS is associated with the presence of morbid obesity as an independent variable. In each category, LOS was longer in the morbidly obese group. IRRs in the nonoperative group were 1.21, 1.25, and 1.36 for pelvic fracture, acetabular fracture, and both pelvic/acetabular fractures, respectively. IRRs in the operative group were 1.01, 1.20, and 1.18 for pelvic fracture, acetabular fracture, and both pelvic/acetabular fractures, respectively. This difference was significant for all categories (*p* < 0.001) except operative pelvic fractures (*p* = 0.785). This follows the same pattern as mean LOS for all categories.

## Discussion

We found that morbid obesity was associated with a significantly higher overall risk of complications in patients with pelvic, acetabular, or combined pelvic/acetabular fractures treated nonoperatively. Similarly, morbidly obese patients with acetabular and combined pelvic/acetabular fractures treated operatively have a significantly higher overall risk of complications compared with the control group. These findings are similar to those of others who found that obese patients (BMI ≥30) have a greater incidence of perioperative complications, including longer operation times [9], greater estimated intraoperative blood loss [10], and higher rates of wound infection and dehiscence, loss of reduction, iatrogenic nerve injury, DVT, pneumonia, and decubitus ulcers [11, 13].

Patients in the morbidly obese group were more likely to sustain fractures from motor vehicle collisions, whereas those in the control group were more likely to have had falls. This means that obese patients’ fractures were more likely to be caused by high-energy mechanisms and correlates with the higher ISS in the obese group. We adjusted for mechanism of injury in our multivariate regression and found that obesity was still associated with higher complication rates.

Not all complications occurred more frequently in the obese group. Rates of deep SSI, pulmonary embolism, wound disruption, and coagulopathy were no higher in the obese group than in the control group. Of the study patients treated operatively, only 1 subset showed greater odds of superficial SSI. Also, only 1 subset of study patients had greater odds of cardiac arrest, with 1 other group having higher odds of bleeding. Some studies have found no increased rates of several perioperative complications in obese or morbidly obese patients. Baldwin et al. [19] found no increased rates of pulmonary embolism, compartment syndrome, or wound infection in 131 morbidly obese patients who sustained lower extremity fractures. Batsis et al. [20] found no increased risk of cardiac complications in 105 obese elderly patients who underwent surgery for hip fractures compared with normal-weight elderly patients. Jiganti et al. [21] found that 103 obese patients who underwent hip or knee arthroplasty did not experience a greater number of days with fever or have higher transfusion rates, greater narcotic needs, or lower hemoglobin levels compared with normal-weight patients. Tucker et al. [22] found no greater risk of complications in 32 obese patients who underwent femoral nailing compared with nonobese patients undergoing the same procedure.

Karunakar et al. [10] found that BMI was a predictor of postoperative complications in 169 patients who underwent open reduction and internal fixation for acetabular fractures. In their study, the authors found that obese patients (BMI ≥30) were 2.1 times as likely to lose more than 750 mL of blood during surgery, 2.6 times as likely to develop DVT, and morbidly obese patients were 5 times as likely to have a wound infection compared with normal-weight patients. Their results are consistent with ours, in that morbidly obese patients who underwent operative treatment of acetabular fractures had significantly higher odds of having a complication.

Porter et al. investigated outcomes of 102 pelvic ring [9] and 41 acetabular [23] injuries in morbidly obese patients. With respect to pelvic ring injuries, the authors found a higher overall complication rate (39% vs. 19%, *p* < 0.001), which was dominated by wound infections. This contrasted with our results, in that rates for overall complications and wound infections in morbidly obese patients who underwent operative fixation were not significantly different than those in control patients. However, there was a higher overall complication rate in morbidly obese patients who underwent nonoperative treatment in our study. Porter et al. [9] also found that morbidly obese patients with pelvic ring injuries also had longer operative times and greater need for subsequent surgical procedures compared with the control group. With respect to acetabular fractures, Porter et al. [23] reported a significantly higher complication rate (relative risk, 2.6), longer operative times, and greater estimated intraoperative blood loss compared with the control group. Again, the complications were primarily related to wound problems. The finding of higher complication rates in morbidly obese patients with acetabular fractures agrees with our findings for nonoperative and operative treatment groups.

In our study, morbidly obese patients had a significantly longer mean LOS compared with control patients in 5 of 6 stratified groups. Porter et al. [23] also found that morbidly obese patients with acetabular fractures had a longer LOS compared with the control group (26 days vs. 15 days, *p* < 0.01). Baldwin et al. [19] found a longer LOS for morbidly obese patients in only 1 of their subcohorts. They did not find increased hospital costs in either cohort of morbidly obese patients; however, they did find that LOS was highly correlated with hospital cost. Fine et al. [24] found that a reduction in LOS could significantly reduce hospital costs.

We found that morbidly obese patients were more likely than their counterparts in the control group to be sent to a rehabilitation facility (*p* < 0.001), which is congruent with the findings of other studies that have examined this parameter in obese patients who underwent emergent or elective surgery of the pelvis or lower extremity [19, 20, 23, 25].

Our study is limited in that participation in the NTDB is voluntary for all hospitals, and the database has few mandatory data fields. Results are limited by quality and accuracy of data entry. The database does not characterize pelvic fractures beyond the location of the injury. The biggest weakness is that there are no specific procedure codes for the treatment of pelvic fractures. It was necessary to extrapolate the treatment of pelvic fractures by eliminating patients who had phalanx or spinal column fractures. This greatly reduced our sample size. The NTDB categorizes patients only as morbidly obese, and not obese, thereby omitting many potential patients.

This is the largest study to our knowledge that examines complications of morbidly obese patients with pelvic and/or acetabular fractures. These data were taken from a nationwide sample, eliminating any geographic or surgeon-based variations or biases that may be present in smaller studies. With an increasing proportion of Americans in the morbidly obese weight group, it is important to be able to discuss the risks of complications associated with nonoperative and operative treatment of pelvic and acetabular injuries and how they may differ according to patient BMI. Because medical treatment is increasingly reimbursed on the basis of injury type and severity [25], it is important for hospitals to be able to bill appropriately for morbidly obese patients to account for higher complication rates and longer hospital stays, which have been shown to correlate with increased hospital costs. Given that so many morbidly obese patients are discharged to care facilities, it would be prudent to prepare these patients for this discharge disposition early in their hospital stay.

## Conclusions

With the exception of pelvic fractures treated operatively, morbidly obese patients had higher rates of complications and longer hospital stays. They were also more likely to be discharged to rehabilitation facilities compared with control patients after pelvic ring or acetabular fracture. Regarding to treatment outcomes and costs, it is important to understand that morbid obesity negatively affects outcomes in operatively treated acetabulum fractures and nonoperatively treated pelvic and acetabulum fractures.
